# 3rd-Degree Atrioventricular Block

**DOI:** 10.21980/J8NP9S

**Published:** 2022-04-15

**Authors:** Patrick Meloy, Dan Rutz, Amit Bhambri

**Affiliations:** * Emory University School of Medicine, Department of Emergency Medicine, Atlanta, GA; ^ University of Wisconsin-Madison School of Medicine and Public Health, Department of Emergency Medicine, Madison, WI; † Swedish Covenant Hospital, Department of Emergency Medicine, Chicago, IL

## Abstract

**Audience:**

This oral boards case is appropriate for emergency medicine residents and medical students on emergency medicine rotations

**Introduction/Background:**

Third-degree heart block (also known as complete heart block) is a cardiovascular emergency that requires prompt recognition. Complete heart block is a type of atrioventricular (AV) block whereby no atrial impulses reach the ventricular conduction system. The most common etiology of AV block is ischemic heart disease, with up to 1 in 5 patients developing some type of conduction disturbance after an MI.^1^ Complete heart block is seen in 8% of patients post-MI.^2^ Other causes include myocarditis, infectious endocarditis, infiltrative cardiac disease, congenital AV blocks, non-ischemic cardiomyopathy, electrolyte disturbances, and drug side effects.^3^ In complete heart block, the heart rate is dependent on the location of the block and a functioning secondary pacemaker within the conduction system. Analysis of the EKG will determine the location of this escape rhythm. For escape rhythms originating at the AV node or high in the His-Purkinje system, the QRS complex will typically be narrow, and the ventricular rate typically in the 40–60 bpm range. For blocks with ventricular escape rhythms, the QRS will appear wide, with rates of 20–40 bpm. Patients presenting with 3rd-degree AVB with ventricular escape rhythms can destabilize. If no escape rhythm generates, patients develop asystole and cardiac arrest. Since 1 in 600 adults over the age of 65 will develop a form of supraventricular conduction abnormality each year, this disease process is important to identify and treat.^4^ Effective management includes accurate interpretation of a 12-lead EKG, assessment of hemodynamic stability and systemic perfusion, and time-sensitive pharmacologic or procedural intervention.

**Educational Objectives:**

At the end of this oral board session, examinees will: 1) demonstrate ability to obtain a complete medical history including detailed cardiac history, 2) demonstrate the ability to perform a detailed physical examination in a patient with cardiac complaints, 3) investigate the broad differential diagnoses which include acute coronary syndrome (ACS), electrolyte imbalances, pulmonary embolism, cerebrovascular accident, aortic dissection and arrhythmias, 4) obtain and interpret the cardiac monitor rhythm strip to identify complete heart block, 5) list the appropriate laboratory and imaging studies to differentiate arrhythmia from other diagnoses (complete blood count, comprehensive metabolic panel, magnesium level, EKG, troponin level, chest radiograph), 6) identify a patient with complete heart block and manage appropriately (administer IV atropine, attempt transcutaneous pacing, place a transvenous pacemaker, emergent consultation with interventional cardiology), 7) provide appropriate disposition to intensive care after consultation with interventional cardiologist.

**Educational Methods:**

This is a straight-forward case which was written to assess learners’ ability to rapidly recognize an unstable cardiac rhythm and to subsequently treat and stabilize the patient. Oral board testing is used as a proxy for the emergency department (ED) and can assist with periodic assessment of resident performance while in the ED.

We have found that oral board testing is a useful tool to assess residents’ critical thinking while still applying pressure that is needed to pass the examination itself. Large groups of residents can be assessed in a short time period without needing to “wait” for a particular clinical condition to present to the ED.

In this case, learners were assessed using a free online evaluation tool, ie, Google forms. Multiple questions were written for each critical action, and the Google form served as the online evaluation and repository. The critical actions of the case were then tied to Emergency Medicine Milestones, and the results were compiled for use during residency clinical competency evaluations. Residents were provided with immediate verbal feedback of their performance and were also given their electronic evaluations when requested.

**Research Methods:**

Learners and instructors were given the opportunity to provide electronic feedback after the case was completed to assess strengths and weaknesses, and subsequent modifications were made. Additionally, learners answered written multiple-choice questions after the case to assess for retention of the material.

**Results:**

Senior learners found this to be a more enjoyable way to refresh their skills than direct lecture. Junior residents and students who encountered this clinical entity first in the oral board rather than in the ED, stated that they enjoyed the ability to “trial run” the case before being faced with an emergent and uncontrolled setting of the ED. Overall, the learners rated the case as 4.7 (1–5 Likert scale, 5 being excellent) after the mock oral board examination was completed.

**Discussion:**

Students and residents who were assessed with a mock oral board session found this to be an improvement over traditional “lecture” and were pleased to have participated. The content is highly relevant to emergency medicine and the format forces learners to be actively engaged in review of the material. The case is a good model for the high stakes testing of written and oral board examinations, and is an effective way to assess a resident’s ability to rapidly assess and manage a life-threatening condition in the ED.

**Topics:**

Third-degree AV block, complete heart block, 3^rd^-degree block, hypotension, syncope, bradycardia, cardiovascular emergency.

## USER GUIDE

**Table:** 

List of Resources:	
Abstract	1
User Guide	4
For Examiner Only	6
Oral Boards Assessment	11
Stimulus	14
Debriefing and Evaluation Pearls	27

**Learner Audience:**

Medical students, interns, junior residents, senior residents, APPs

**Time Required for Implementation:**

Case: 15 minutes as a single case

Debriefing: 10 minutes

**Learners per instructor:**

1

**Topics:**

Third-degree AV block, complete heart block, 3^rd^-degree block, hypotension, syncope, bradycardia, cardiovascular emergency.

**Objectives:**

By the end of this oral boards case, examinees will be able to:

1. Demonstrate ability to obtain a complete medical history including detailed cardiac history
2. Demonstrate the ability to perform a detailed physical examination in a patient with cardiac complaints
3. Investigate the broad differential diagnoses which include acute coronary syndrome (ACS), electrolyte imbalances, pulmonary embolism, cerebrovascular accident, aortic dissection and arrhythmias
4. Obtain and interpret the cardiac monitor rhythm strip to identify complete heart block
5. List the appropriate laboratory and imaging studies to differentiate arrhythmia from other diagnoses (complete blood count, comprehensive metabolic panel, magnesium level, EKG, troponin level, chest radiograph)
6. Identify a patient with complete heart block and manage appropriately (administer IV atropine, attempt transcutaneous pacing, place a transvenous pacemaker, emergent consultation with interventional cardiology)
7. Provide appropriate disposition to intensive care after consultation with interventional cardiologist

### Linked objectives and methods

The learner in this case must be able to synthesize available history, physical examination and cardiac monitor findings (Objectives 1 and 2) in order to develop a broad differential of a patient who is in complete heart block (Objective 3). Without interpreting the cardiac monitor, the diagnosis may be missed if the learner does not identify the patient in complete heart block (Objective 3, 4 and 5). The oral board formatting allows the learner to interpret the rhythm strip in real-time in order to identify a life-threatening arrhythmia (Objective 4). The learner must be able to identify complete heart block and provide timely and appropriate treatment and disposition to prevent an adverse outcome (Objective 6 and 7). Debriefing of the case immediately afterward ensures assimilation of the sources of data to obtain the correct diagnosis and appropriate management of the case.

### Recommended pre-reading for instructor

- None, review references as needed

### Results and tips for successful implementation

This case was best used as an oral board examination. The learner should be directly observed by the examiner, either in-person or by video, and additional learners or instructors can also be present to observe. Learners were tested during emergency medicine conferences and during a mock oral board examination and oral board practice sessions. Assessment forms were created for the case using Google forms (http://docs.google.com/forms), and these were tied to Emergency Medicine Milestones (https://www.acgme.org/Portals/0/PDFs/Milestones/EmergencyMedicineMilestones.pdf?ver=2015-11-06-120531-877). Using this method, the oral board formatting could assess residents’ clinical ability to practice in a non-threatening environment but also evaluate their progress along the ACGME’s milestones.

It was a challenging case for medical students and interns, and tests the efficient, higher-level processing needed for senior residents. Fifty-five learners performed the examination and were evaluated. Most learners were able to obtain the diagnosis; however, there were some challenges in appropriate management of the case. The case was initially trialed with an electrolyte abnormality, ie, severe hypomagnesemia, though this caused too much additional time to treat and evaluate, was deemed not essential to the case, and was removed.

After a mock oral board session was completed, learners were given the ability to rate the cases individually, and this case scored 4.7 (1–5 Likert scale, 5 being excellent). Learners stated that the oral board session was a “quick diagnosis but required me to think about how to investigate for underlying causes,” and “hard to guess right off the bat, scary that I needed to resuscitate, but glad I finished the case.” This is a highly testable concept in emergency medicine; we feel this case is an important addition to any written or oral board session.

## Figures and Tables

**Table:** 

**Vitals:**	HR 44	BP 92/56	RR 22	Temp 36.7°C	O_2_Sat 100% (2-L nasal canula)

**Table:** 

	Milestone	Did not achieve level 1	Level 1	Level 2	Level 3
**1**	**Emergency Stabilization (PC1)**	□ **Did not achieve Level 1**	□ **Recognizes abnormal vital signs**	□ **Recognizes an unstable patient, requiring intervention** **Performs primary assessment** **Discerns data to formulate a diagnostic impression/plan**	□ **Manages and prioritizes critical actions in a critically ill patient** **Reassesses after implementing a stabilizing intervention**
**2**	**Performance of focused history and physical (PC2)**	□ **Did not achieve Level 1**	□ **Performs a reliable, comprehensive history and physical exam**	□ **Performs and communicates a focused history and physical exam based on chief complaint and urgent issues**	□ **Prioritizes essential components of history and physical exam given dynamic circumstances**
**3**	**Diagnostic studies (PC3)**	□ **Did not achieve Level 1**	□ **Determines the necessity of diagnostic studies**	□ **Orders appropriate diagnostic studies** **Performs appropriate bedside diagnostic studies/procedures**	□ **Prioritizes essential testing** **Interprets results of diagnostic studies** **Considers risks, benefits, contraindications, and alternatives to a diagnostic study or procedure**
**4**	**Diagnosis (PC4)**	□ **Did not achieve Level 1**	□ **Considers a list of potential diagnoses**	□ **Considers an appropriate list of potential diagnosis** **May or may not make correct diagnosis**	□ **Makes the appropriate diagnosis** **Considers other potential diagnoses, avoiding premature closure**
**5**	**Pharmacotherapy (PC5)**	□ **Did not achieve Level 1**	□ **Asks patient for drug allergies**	□ **Selects an appropriate medication for therapeutic intervention, considering potential adverse effects**	□ **Selects the most appropriate medication(s) and understands mechanism of action, effect, and potential side effects** **Considers and recognizes drug-drug interactions**
**6**	**Observation and reassessment (PC6)**	□ **Did not achieve Level 1**	□ **Reevaluates patient at least one time during the case**	□ **Reevaluates patient after most therapeutic interventions**	□ **Consistently evaluates the effectiveness of therapies at appropriate intervals**
**7**	**Disposition (PC7)**	□ **Did not achieve Level 1**	□ **Appropriately selects whether to admit or discharge the patient**	□ **Appropriately selects whether to admit or discharge** **Involves the expertise of some of the appropriate specialists**	□ **Educates the patient appropriately about their disposition** **Assigns patient to an appropriate level of care (ICU/Tele/Floor)** **Involves expertise of all appropriate specialists**
**22**	**Patient centered communication (ICS1)**	□ **Did not achieve level 1**	□ **Establishes rapport and demonstrates empathy to patient (and family)** **Listens effectively**	□ **Elicits patient’s reason for seeking health care**	□ **Manages patient expectations in a manner that minimizes potential for stress, conflict, and misunderstanding**.
**23**	**Team management (ICS2)**	□ **Did not achieve level 1**	□ **Recognizes other members of the patient care team during case (nurse, techs)**	□ **Communicates pertinent information to other healthcare colleagues**	□ **Communicates a clear, succinct, and appropriate handoff with specialists and other colleagues** **Communicates effectively with ancillary staff**

**Table:** 

**#1**	**Patient Information Form**
**#2**	**Arterial Blood Gas**
**#3**	**CBC**
**#4**	**BMP**
**#5**	**Troponin**
**#6**	**Coagulation Panel**
**#7**	**Urinalysis**
**#8**	**CXR**
**#9**	**Monitor Rhythm Strip**
**#10**	**Initial EKG**
**#11**	**Repeat EKG (Ventricular tachycardia)**
**#12**	**Repeat Monitor Rhythm Strip (ventricular tachycardia)**

**Table:** 

**Patient’s Name:**	**Jeanie Johnson**
**Age:**	**58**
**Gender:**	**Female**
**Chief Complaint:**	**Syncope**
**Person Providing History:**	**Patient**
**Vital Signs:**	
**Temp:**	**36.7°C**
**BP:**	**92/56**
**P:**	**44**
**RR:**	**22**
**Pulse Ox:**	**100% (2-liters nasal cannula)**
**Weight:**	**127 kg**

**Table:** 

**pH**	**7.37**
**pCO2**	**38 mmHg**
**pO2**	**94 mmHg**
**HCO3**	**22 mmol/L**
**O2 sat**	**100%**

**Table:** 

**White blood cell count (WBC)**	**5.7 x1000/mm3**
**Hemoglobin (Hgb)**	**11.2 g/dL**
**Hematocrit (Hct)**	**42%**
**Platelets**	**291 ×1000/mm3**
**Neutrophils**	**64%**
**Lymphocytes**	**24%**
**Monocytes**	**5%**
**Eosinophils**	**4%**
**Basophils**	**1%**

**Table:** 

**Sodium**	**140 mEq/L**
**Potassium**	**3.6 mEq/L**
**Chloride**	**104 mEq/L**
**Bicarbonate**	**23 mEq/L**
**Blood Urea Nitrogen (BUN)**	**15 mg/dL**
**Creatinine (Cr)**	**1.2 mg/dL**
**Glucose**	**191 mg/dL**

**Table:** 

**Troponin**	**0.10 mcg/L (ref <0.05 mcg/L)**

**Table:** 

**Prothrombin Time (PT)**	**29.1 seconds**
**Partial Thromboplastin Time (PTT)**	**15.1 seconds**
**International Normalized Ratio (INR)**	**1.1**

**Table:** 

**Color**	**Yellow**
**Appearance**	**Clear**
**Sp Gravity**	**1.020**
**pH**	**6.4**
**Glucose**	**Trace**
**Protein**	**1+**
**Ketones**	**Negative**
**Leukocyte Esterase**	**Negative**
**Nitrite**	**Negative**
**Blood**	**Negative**
**Microscopy**	
**White blood cells (WBC)**	**Negative**
**Red blood cells (RBC)**	**Negative**
**Squamous cells**	**5/hpf**
**Bacteria**	**0–2/hpf**

**Figure:**
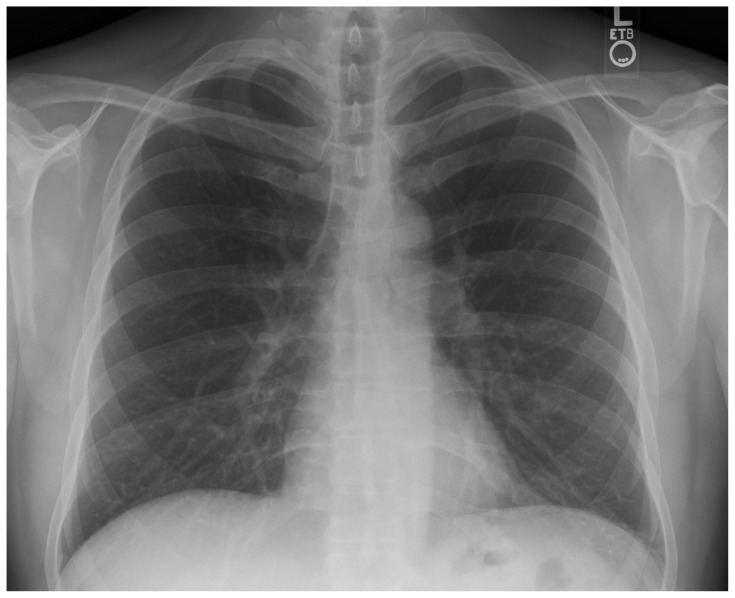
Image Source: Stillwaterising. Normal PA chest radiograph. In: Wikimedia Commons. https://commons.wikimedia.org/wiki/File:Chest_Xray_PA_3-8-2010.png. Published March, 2008. Accessed October 1, 2021. Public Domain.

**Figure:**
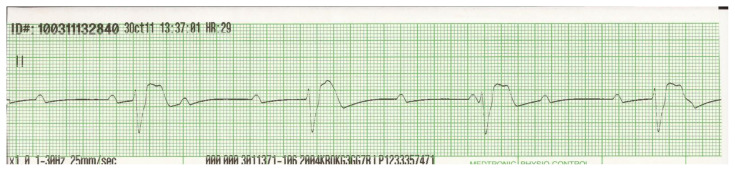
Image Citation: Author’s own image

**Figure:**
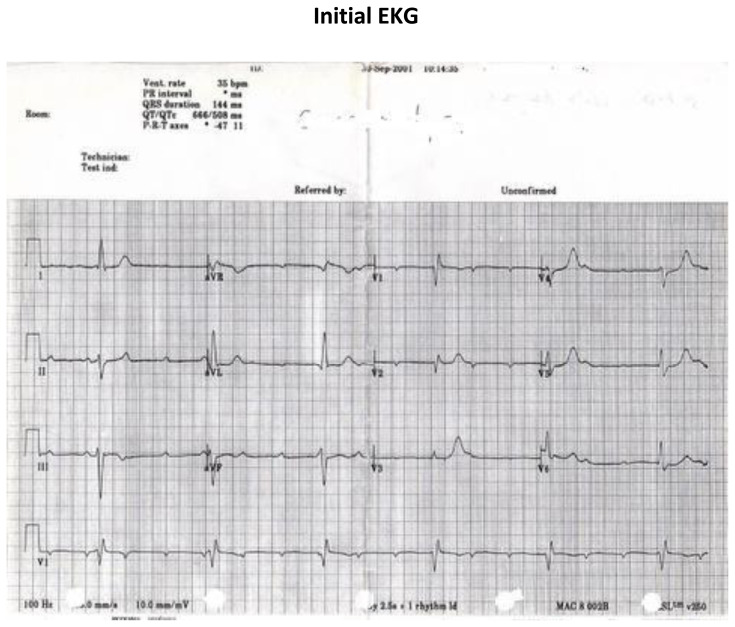
Image Citation: Author’s own image

**Figure:**
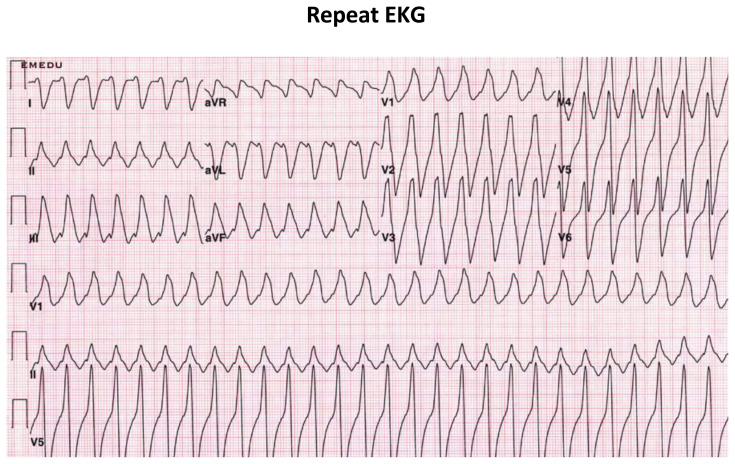
Image Citation: Author’s own image

**Figure:**
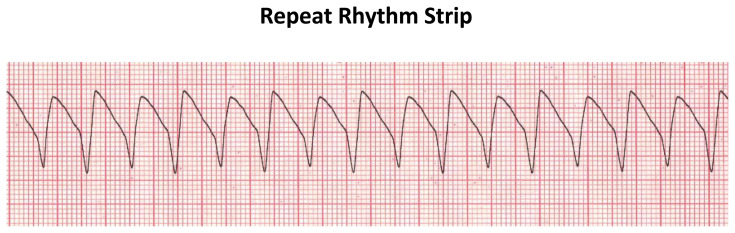
Image Citation: Author’s own image

## FOR EXAMINER ONLY

### Oral Case Summary

#### Diagnosis: 3rd-Degree (Complete) Heart Block

**Case Summary:** This is a 58-year-old female patient with a previous history of hypertension and diabetes who presents to the emergency department after a syncopal episode. She is complaining of substernal chest pain upon arrival, stating that it is sharp and radiating to the right shoulder and has been present for around 24 hours. She thought it might be indigestion, but today she felt dizzy and fainted after getting up from the couch, which prompted her to activate EMS. She is initially bradycardic and hypotensive and should be brought into the resuscitation room upon arrival.

**Order of Case:** This is the case of a female patient presenting to the ED with syncope, who is found to have a 3rd-degree heart block. The diagnosis should be apparent via the initial rhythm-strip and/or EKG. The patient should immediately have transcutaneous pacing pads placed and preparations should be made for a transvenous pacemaker in the ED. If given, atropine will only temporarily correct the bradycardia, but will not affect the blood pressure, and again, is only temporary. If the initial rhythm is not recognized, the patient will deteriorate into symptomatic ventricular tachycardia, requiring the use of advanced cardiac life support (ACLS) resuscitation. Return of spontaneous circulation (ROSC) should be obtained and the 3rd-degree should again be recognized. Learners may intubate the patient at this point, which is acceptable, but is not the testable concept. Once the block is appreciated, the patient should be admitted to the intensive care unit, and interventional cardiology should be consulted to assess for permanent pacemaker placement.

If the learner does not appreciate the 3rd-degree block, ROSC can still be obtained, and the patient can still be admitted to the ICU. The case will not be passed, but the learner should be debriefed promptly and will be given the diagnosis and the appropriate EKG findings.

**Disposition:** Admit to intensive care unit

**Critical Actions:**

1. Triage the patient to the resuscitation room
2. Request the patient be placed on the cardiac monitor, obtain a rhythm strip/EKG and interpret the findings
3. Place temporary transcutaneous pacemaker pads
4. Insert transvenous pacemaker
5. Admit to intensive care unit with interventional cardiology consult

### Historical Information

**Chief Complaint:** Syncope

**History of present illness:** This is a 58-year-old female patient with previous history of coronary artery disease, myocardial infarction, hypertension, and diabetes, who presents to the ED via EMS after experiencing a syncopal event. She was at home when the event happened, in a chair, and did not fall to the ground or strike her head. She states now that she is awake, she is experiencing substernal chest pain that is radiating into the right shoulder. She states that she has been having generalized weakness and exertional dyspnea for the last 24 hours. She has had episodes of diaphoresis. She denies fevers or chills, nausea or vomiting.

**Past Medical history:** HTN, DM, CAD, MI

**Past Surgical history:** Appendectomy, cholecystectomy, carpal tunnel release

**Patient’s Medications:** Amlodipine, aspirin, metformin, carvedilol

**Allergies:** Penicillin and shellfish

**Social history:**

- Tobacco: Current, 1 ppd
- Alcohol: Denies
- Drug use: Denies

**Family history:** Does not recall

### Physical Exam Information

**Weight:** 127 kg

**General appearance:** Disheveled, mild-to-moderate distress

**Primary survey:**

- **Airway:** Intact
- **Breathing:** Intact, tachypneic
- **Circulation:** Intact peripheral pulses, hypotensive, bradycardic

**Physical examination:**

- **General appearance:** Disheveled, mild-to-moderate distress
- **Head, eyes, ears, nose and throat (HEENT):**
  ○ **Head:** Within normal limits
  ○ **Eyes:** Within normal limits
  ○ **Ears:** Within normal limits
  ○ **Nose:** Within normal limits
  ○ **Oropharynx/Throat:** Within normal limits
- **Neck:** No JVD, trachea midline
- **Chest:** Tachypneic, lungs CTA bilaterally, no wheezes, crackles or rales
- **Cardiovascular:** Bradycardic, irregular heart tones, no murmurs, 1+ radial and dorsalis pedis pulses bilaterally
- **Abdominal/GI:** Within normal limits
- **Genitourinary:** Deferred
- **Rectal:** Within normal limits
- **Extremities:** Trace pitting edema of the bilateral lower extremities, 2+ radial and dorsalis pulses bilaterally
- **Back:** Within normal limits
- **Neuro:** Within normal limits
- **Skin:** Diaphoretic
- **Lymph:** Within normal limits
- **Psych:** Within normal limits

### Critical Actions and Cueing Guidelines

1. **Critical Action 1:** *Triage the patient to the resuscitation room*
  Cueing Guideline (if applicable): RN to ask, “Do you think this patient needs advanced monitoring? Should she be closer to the nursing station?”
2. **Critical Action 2:** *Request the patient be placed on the cardiac monitor, obtain an immediate rhythm-strip/EKG and interpret the findings*.
  Cueing Guideline (if applicable): RN/student to ask, “Do you want me to hook her up to the cardiac monitor before we place an IV?”
3. **Critical Action 3:** *Place temporary transcutaneous pacemaker*.
  Cueing Guideline (if applicable): RN to ask, “Do you want me to get out the portable monitor in case she needs to be shocked?”
4. **Critical Action 4:** *Insert transvenous pacemaker*.
  Cueing Guideline (if applicable): RN to ask, “Are you sure the patient is stable enough to be transported through the hospital to the ICU on the pacer-pads?”
5. **Critical Action 5:** *Admit to hospital intensive care unit with interventional cardiology, (or electrophysiology) consultation*.
  Cueing Guideline (if applicable): Accepting to ask, “What does cardiology plan to do for this?”

## ORAL BOARDS ASSESSMENT

*3* *^rd^* *-Degree Atrioventricular Block*

Learner: _________________________________________

**Critical Actions:**

□ Triage the patient to the resuscitation room
□ Request the patient be placed on the cardiac monitor, obtain a rhythm strip/EKG and interpret the findings
□ Place temporary transcutaneous pacemaker pads
□ Insert transvenous pacemaker
□ Admit to intensive care unit with interventional cardiology consult

**Summative and formative comments:**

**Milestone assessment:**

## DEBRIEFING AND EVALUATION PEARLS

### 3^rd^-Degree Atrioventricular Block

1. Third-degree heart block occurs when no atrial electrical impulses reach the ventricular conduction system. It is a cardiovascular emergency.
2. Common causes of 3rd-degree heart block include acute coronary syndrome, cardiomyopathy, electrolyte derangements (particularly hyperkalemia), infiltrative cardiac illnesses, and medication side effects.^2^
3. Diagnosis of 3rd-degree heart block is made via interpretation of a 12-lead EKG or a cardiac rhythm strip. The key feature of 3rd-degree heart block is AV dissociation, whereby atrial and ventricular impulses appear independent of each other.^3^
4. When electrical impulses are interrupted, ectopic pacemaker cells can sustain the heart rate, with the rate of spontaneous depolarization decreasing further down the conduction pathway (SA Node → Atria → AV Node → Ventricles). In the usual state, the slower pacemaker cells are superseded by impulses from above.^3^,^5^
5. In 3rd-degree heart block, subsidiary pacer cells will drive the perfusing heart rate. Therefore, the more distal the conduction block, the slower the heart rate will be.^1^
6. Junctional escape rhythms originate near the AV-node and will appear as narrow-complex QRS impulses with rates typically in the 40–60s.^3^,^5^
7. Ventricular escape rhythms originate below the AV-node and will appear as wide-complex QRS impulses with rates in the 20s–40s.^3^,^5^
8. Treatment of 3rd-degree heart block includes stabilization via transcutaneous pacing. Pacer pads should be placed on the chest and connected to an impulse generator. Starting at 80mA and a rate of 80–100 beats per minute, providers should adjust the energy to the lowest that initiates pacing. If tolerable given patient hemodynamics, pain control and sedation are considerations as well.^6^
9. If transcutaneous pacing fails, providers should administer epinephrine 20–50 ug IV bolus and epinephrine 2–10 ug/min infusion while placing transvenous pacer.^6^
10. Transvenous pacing is indicated when transcutaneous pacing fails to improve heart rate and blood pressure in 3rd-degree AV-block.^6^
11. Reversible causes of 3rd-degree AV-block should be treated if contributory (ACS, hyperkalemia, drug overdose).^6^
12. For 3rd-degree AV-block due to hyperkalemia, administer IV Calcium (3g Calcium Gluconate via peripheral IV or 1g Calcium Chloride via central line), 5 units regular insulin, two ampules of D50, and 10–20mg nebulized Albuterol. Calcium can be re-dosed at 5 minutes if there is no change in the EKG, or there is brief improvement followed by block recurrence. If unsuccessful in terminating block, temporize with transcutaneous or transvenous pacing and arrange for emergent hemodialysis.^10^
13. For 3rd-degree AV-block due to beta blocker or calcium channel blocker overdose, administer IV Calcium (3g Calcium Gluconate via peripheral IV or 1 g Calcium Chloride via Central Line), 5 mg IV Glucagon bolus, and initiate IV Insulin infusion 1 unit/kg/hr with dextrose infusion.^11^
